# Axial length growth difference between eyes after monocular laser refractive surgery: eight patients who underwent myopic laser ablation for both eyes at intervals of several years

**DOI:** 10.1186/s12886-022-02243-y

**Published:** 2022-01-11

**Authors:** Chenghai Weng, Fei Xia, Dang Xu, Xingtao Zhou, Liangcheng Wu

**Affiliations:** 1 Department of Ophthalmology, Jing-An District Center Hospital of Shanghai (Huashan Hospital Fudan University Jing-An Branch), Shanghai Medical College, Fudan University, No.259 Xikang Road, Shanghai, 200031 China; 2 Eye Institute and Department of Ophthalmology, Eye & ENT Hospital, Fudan University, No.19 Baoqing Road, Shanghai, 200031 China; 3 NHC Key Laboratory of Myopia (Fudan University), Key Laboratory of Myopia, Chinese Academy of Medical Sciences, Shanghai, China; 4 Shanghai Research Center of Ophthalmology and Optometry, Shanghai, China

## Abstract

**Background:**

Myopia is a global public health issue. Controlling myopia progression is a primary focus of myopia studies today. Peripheral retinal myopic defocus is considered the mechanism for reduced myopia progression in orthokeratology studies. The topographic change in the front corneal surface after laser refractive surgery and orthokeratology procedures may appear similar. The purpose of this study was to explore the role of myopic laser ablation on axial length (AL) growth.

**Methods:**

Myopic patients who underwent monocular excimer laser refractive surgery first in one eye and then in another eye several years later because of myopia occurrence or myopia progression were recruited. The axial length elongation and refraction (spherical equivalent) between the two eyes were observed and compared.

**Results:**

A total of 8 myopic patients were enrolled in the study. The AL increased from 24.52 ± 0.96 mm to 24.68 ± 1.03 mm but without significance (T = 1.49, *P* > 0.05) in the ablated eyes. The AL increased significantly from 23.73 ± 0.91 mm to 24.26 ± 0.95 mm in the nonablated eyes (T = 6.76, *P* < 0.001). The AL elongation of the ablated eyes with 0.16 ± 0.30 mm growth was significantly lower than that of the nonablated eyes with 0.53 ± 0.32 mm growth (T = 8.98, *P* < 0.001). The spherical equivalent (SE) increased significantly in the ablated eyes (− 0.59 ± 0.21 (D), T = 6.36, P < 0.001) and in the nonablated eyes (− 0.97 ± 0.55 (D), T = 4.91, *P* < 0.01), and the difference between the two eyes was significant (T = 3.05, *P* < 0.05).

**Conclusions:**

The inhibitory effect of myopic laser ablation on AL elongation reported in the limited case studies argues for animal research on its efficacy as a new intervention for myopia progression.

## Background

The prevalence rate of myopia, which is an extremely common eye disorder worldwide, especially in East Asian countries, has increased to 70–90% [1]. In 2050, a total of 4.758 billion people worldwide (49.8% of the world’s population) are expected to have myopia, and 938 million people (9.8% of the world’s population) are expected to experience high myopia [2]. Myopia is rapidly becoming a global public health issue, with myopia-related complications being the major causes of visual impairments and blindness [3, 4]. Methods to control myopia progression is always a major focus of myopia studies. Multiple studies have shown that orthokeratology can slow myopia progression in paediatric patients [5–8]. The mechanism for reduced myopia progression is most likely due to the presence of sustained peripheral retinal myopic defocus [9, 10].

Excimer laser refractive surgeries, including laser-assisted in situ keratomileusis (LASIK), photorefractive keratectomy (PRK) or laser epithelial keratomileusis (LASEK), reduce the central area’s curvature by reshaping the front surface of the cornea to treat myopia. As a result, the corneal periphery is steeper than the central area, and the rays near the optical axis focus behind the peripheral rays [11]. Many studies have shown that topographic changes in the front corneal surface after laser refractive surgery and orthokeratology procedures may appear similar [12, 13]. Therefore, we hypothesize that myopic refractive corneal surgery can also play a role similar to orthokeratology contact lenses (OK lenses) in slowing myopia progression. Considering the contribution of axial length (AL), lens power, and corneal power together with multiple linear regression analyses, AL can explain up to 96% of the variation in refraction in populations [14]. The AL is a valid parameter to monitor myopic progression. In our study, AL was used to evaluate myopia progression. In the present study, we collected the data of adolescents who underwent single eye myopic ablation and then again in another eye several years later because of myopia occurrence or myopia progression. The aim of the study was to explore the role of myopic refractive surgery in slowing myopia progression.

## Methods

Patients with myopia who underwent monocular myopic laser surgery in one eye and then underwent surgery in another eye several years later because of myopia occurrence or myopia progression in three practical clinic centres during 2007-2017 (Jing-An district center hospital, Shanghai; Beng-Bu Peace Eye Hospital, Anhui Province; Yi-Xing Eye Hospital, Jiangsu Province) were enrolled in this study. All patients provided written informed consent. Moreover, for patients under 18 years old, informed consent was obtained from their parents before surgery. The study was performed in accordance with the Declaration of Helsinki for research involving human subjects. The study met all standards for ethical approval in China, and the protocol was approved by the institutional board at Jing-An District Center Hospital.

A comprehensive preoperative and postoperative ocular examination was performed in each eye, including visual acuity, best visual acuity, refraction, topography, corneal thickness and axial length, before both refractive surgeries. Refraction was measured under cycloplegia and using the fogging technique (high plus) for both eyes in preoperative examination and using the fogging technique in postoperative examination. Manifest refraction for both eyes between 3 months after surgery in ablated eyes and at preoperative examination in nonablated eyes was compared. The AL of both eyes was measured using the same type A scan (SW-1200, Suowei Electronic Technology Co., Ltd., Tianjin, China) in two preoperative examinations in three centres. Myopic refractive ablation was performed by the same surgeon (WU) using a LaserSight LSX (LaserSight Technologies, Inc., USA) in Yi-Xing Eye Hospital, an AOV excimer laser (66 vision Co., Ltd., Suzhou, China) in Beng-Bu Peace Eye Hospital and an Esiris excimer laser (Schwind Eye Tech Solutions, Kleinostheim, Germany) in Jing-An District Center Hospital.

AL elongation and SE changes were observed and compared between the two eyes.

All statistical analyses were performed using Excel® [Microsoft Inc., USA] and SPSS for ^Windows^® v11.0 [IBM, USA]. All data are presented as the mean ± standard deviation (SD). A paired t test (two-tailed) was used to analyse the changes in SE and AL. A *P* value of less than 0.05 was deemed to be statistically significant.

## Results

Approximately 1995 of over 9000 patients between 17 and 18 years of age underwent myopic refractive surgery, eighty-seven patients underwent monocular surgery, and only eight patients underwent treatment of the other eye. The other 79 patients had stable vision and refraction in the follow-up period. The details of all eight patients (6 males, 2 females) are illustrated in Table 1. The average age was 17.63 ± 0.52 years old at the time of the initial surgical procedure, with a follow-up time of approximately 3.79 ± 0.80 years. All surgical procedures were based on LASEK.

**Table 1:** SE and AL changes in each patient

Patients	Age	Eye	Group	SE0(D)	AL1 (mm)	SE1(D)	SE2(D)	AL2(mm)	Optical correction in nonablated eyes	Interval(yrs)
1	17	OD	ablated	-2.5	24.86	0.5	0	24.9		3.5
		OS	Nonablated	-0.5	23.94	-0.5	-1.75	24.5	Full time-CL	
2	18	OD	ablated	-1.75	23.28	0.25	0	23.35		4.5
		OS	Nonablated	-0.5	22.56	-0.5	-1.25	23.25	Part time-CL	
3	18	OD	Nonablated	-0.5	22.08	-0.5	-1	22.56	Part time-CL	2.8
		OS	Ablated	-2	22.78	0.5	0.25	22.83		
4	17	OD	Nonablated	-0.25	24.43	-0.25	-1	25.03	No-CL	4.5
		OS	Ablated	-1.75	25.36	0.5	0	25.43		
5	18	OD	Ablated	-2	24.81	0.5	-0.25	24.86		3.3
		OS	Nonablated	-0.5	24.02	-0.5	-1.25	24.47	No-CL	
6	18	OD	Ablated	-1.75	24.73	0.25	-0.5	25.64		5.1
		OS	Nonablated	-0.5	24.17	-0.5	-2.75	25.31	Full time-CL	
7	17	OD	Ablated	-2	25.35	0.75	0.25	25.41		3.2
		OS	Nonablated	-0.5	24.66	-0.25	-1	24.97	Part time-CL	
8	18	OD	Nonablated	-0.25	24.01	-0.25	-1	24.02	NO-CL	3.4
		OS	Ablated	-2	24.99	0.5	0.25	25.02		

*SE* spherical equivalent, *AL* axial length, *SE0* spherical equivalent in the preoperative examination in ablative eye, *SE1* spherical equivalent in the 3months after first eye ablation, *SE2* spherical equivalent in the preoperative examination in another eye, *AL1* axial length in the preoperative examination in ablative eye, *AL2* axial length in the preoperative examination in another eye, *CL* contact lens

The AL increased from 24.52 ± 0.96 mm to 24.68 ± 1.03 mm and remained relatively stable in the ablated eyes (T = 1.49, *P* > 0.05). The AL increased significantly from 23.73 ± 0.91 mm to 24.26 ± 0.95 mm in the nonablated eyes (T = 6.76, *P* < 0.001). The AL elongation was 0.16 ± 0.30 mm in the ablated eyes and 0.53 ± 0.32 mm in the nonablated eyes. The axial growth in the ablated eyes was significantly lower than that in the nonablated eyes (T = 8.98, *P* < 0.001).

The SE change is shown in Tables 1 and 2. The SE increased significantly in ablated eyes (− 0.59 ± 0.21 (D), T = 6.36, P < 0.001) and in nonablated eyes (− 0.97 ± 0.55 (D), T = 4.91, *P* < 0.01), and the difference between the two eyes was significant (T = 3.05, *P* < 0.05).

**Table 2:** SE and AL in ablated eyes and nonablated eyes

	Ablated eyes	Nonablated eyes
Age (yrs)	17.63±0.52	
Follow-time(yrs)	3.79±0.80	
SE1(D)	0.47±0.16	-0.41±0.13
Initial AL(mm)	24.52±0.96	23.73±0.91
SE increase(D)	-0.59±0.21^*^	-0.97±0.56^*✱^
AL elongation(mm)	0.16±0.30	0.53±0.32^*✱^

*SE* spherical equivalent, *SE1* spherical equivalent at 3 month after myopic ablation in first eye, *AL* axial length;

^*^P<0.05, ^**✱**^comparison between ablated eyes and nonablated eyes

## Discussion

The mechanism of myopia aetiology and progression is very complex. Many risk factors that can induce myopia have been identified, including genetic factors, near work, insufficient light exposure, lack of physical activity, diet, a higher level of education, urbanization, corneal astigmatism, internal astigmatism and accommodation lag [15, 16]. Presently, therapies to manage myopia progression in teenagers are mainly limited to atropine, wearing OK lens, and promoting outdoor activities.

Many studies have confirmed that the OK lens can control myopia progression in school-age children [5–8, 13, 17]. The refractive power in the centre of the cornea is reduced and increases in the peripheral zone after OK lens correction, thereby allowing a reduction in hyperopia defocus of the peripheral retina. Peripheral hyperopia defocus is deemed to be one of the mechanisms of myopia progression in juveniles [17, 18]. Hyperopic defocus of the peripheral retina has also been postulated as a risk factor for myopia in humans. Uncorrected myopic eyes generally exhibit hyperopic relative peripheral refraction (RPR) in the horizontal ocular meridian, while uncorrected hyperopic eyes typically exhibit myopic RPR [18, 19]. Experiments in primates have provided convincing evidence that drastic variations in peripheral defocus influence axial eye growth and emmetropization [20]. The myopic shift in peripheral retinal defocus caused by orthokeratology has been hypothesized to cause a reduction in axial growth [21].

It is well known that laser myopia refractive surgeries also alter the corneal contour in a similar manner to wearing OK lens. The topographic changes that occur at the front corneal surface after orthokeratology and excimer laser refractive surgery appear similar when looking at the curvature maps. The central corneal contour became flatter, and the periphery became steeper. As a result, the corneal periphery is steeper than the central area, and the rays near the optical axis focus behind the peripheral rays [9–11]. A myopic defocus in the peripheral retina may be produced by laser refractive surgery. Whether myopia refractive ablation can control the progression of myopia has not yet been reported. Since almost all patients with myopia who undergo refractive surgery are over 18 years old, the refractions are typically stable. In addition, most patients are subjected to binocular surgery. It is hard to prove that myopia refractive corneal ablation can control myopia progression. However, in practice, it is impossible to ensure the stability of myopia for every patient before refractive surgery, especially for younger patients. In our study, we collected data from only eight patients who underwent such monocular refractive surgical procedures among 87 patients across the three clinical centres. Eight patients asked for refractive surgery in another eye because of myopia progression or occurrence, and the other 79 patients had stable vision and refraction in the follow-up period. We compared the difference in axial growth in both eyes to explore the possible mechanism of myopia control. Theoretically, hyperopic RPR can be controlled in ablated eyes. Hyperopic RPR still existed after refractive correction using a contact lens in nonablated eyes. The present study showed that the increase in AL was 0.16 ± 0.30 mm in the ablated eyes versus 0.53 ± 0.32 mm in the nonablated eyes, and the difference was statistically significant (T = 8.98, *P* < 0.001). The study also showed that the SE increase in ablated eyes was significantly lower than that in nonablated eyes (T = 3.05, *P* < 0.05). Therefore, we speculate that myopic corneal ablation may help to control myopia progression.

However, the changes in the elevation profile of the anterior corneal surface were remarkably different between surgical and OK lens treatments. The mechanism that drives the increase in corneal power at the peripheral corneal zones was also different between the two treatments [10, 11]. There may be other mechanisms for controlling myopia progression in the study.

We admit there are many limitations in the study. First, the AL was tested using an applanation ultrascan technique in the study with a limited precision of approximately 100 μm and accuracy that is affected by varying amounts of corneal indentation by the ultrasound probe. Second, the sample size in this study was too small. We only collected data from eight patients whose refraction appeared unstable and who underwent another eye refractive ablation at intervals of several years from 87 patients who underwent monocular myopic refractive surgery. We did not observe AL changes in the other 79 patients. Third, peripheral refraction was not tested in these cases. Finally, limited to the ethics requirements, we also cannot conduct a clinical study in which myopic ablation slows myopia progression in school-age children. Relevant animal studies should be conducted to explore the role of myopic ablation on myopia progression.

## Conclusions

The inhibitory effect of myopic laser ablation on AL elongation reported in the limited case studies argues for animal research on its efficacy as a new intervention for myopia progression.

## Data Availability

The datasets used and/or analyzed during the current study available from the corresponding author on reasonable request.

## Abbreviations

AL: Axial lenghth
SE: Spherical equivalent
OK lens: Orthokeratology contact lenses
RPR: Relative peripheral refraction
LASEK: Laser subepithelial keratomileusis
LASIK: Laser-assisted in situ keratomileusis
PRK: Photorefractive keratectomy
CL: Contact lens
